# Tolerance for ambiguity, reappraisal, and suppression mediate the relationship between problematic internet use and procrastination

**DOI:** 10.1007/s12144-022-03745-0

**Published:** 2022-10-11

**Authors:** Seyed Javad Emadi Chashmi, Jafar Hasani, Daria J. Kuss, Mark D. Griffiths, Fatemeh Shahrajabian

**Affiliations:** 1 Department of Clinical Psychology, Shahid Beheshti University, Tehran, Iran; 2 Department of Clinical Psychology, Kharazmi University, Tehran, Iran; 3 International Gaming Research Unit, Psychology Department, Nottingham Trent University, 50 Shakespeare Street, Nottingham, NG1 4FQ UK

**Keywords:** Problematic internet use, Procrastination, Tolerance for ambiguity, Reappraisal, Suppression

## Abstract

The current scientific literature lacks studies on the relationship between problematic internet use (PIU) and procrastination, especially regarding the mediating mechanisms underlying this relationship. The present study examined the association between procrastination and PIU, as well as determining the mediating roles of tolerance for ambiguity, reappraisal, and suppression. The conceptual model was tested using data collected from 434 Iranian college students. The participants completed a number of psychometric scales assessing procrastination, PIU, tolerance for ambiguity, reappraisal, and suppression. Structural equation modeling was used to test the hypothesized model. Results showed that PIU, tolerance for ambiguity, and suppression were positively associated with procrastination, and that there was a negative association between reappraisal and procrastination. Moreover, the mediation analysis indicated that tolerance for ambiguity, reappraisal, and suppression fully mediated the association between PIU and procrastination. However, it is also possible to interpret the results as suggesting that PIU is unimportant as a predictor for procrastination once mediators are controlled for.

## Introduction

### Background

In recent years, electronic learning has been introduced into schooling and is changing the context of pedagogy with increasing access to devices, the internet, online learning environments and collaboration tools (Selwyn et al., 2017), resulting in varying degrees of integration and infusion of digital technology within schooling systems (Starkey, 2019). Online learning increased significantly in pedagogical environments when the COVID-19 pandemic impacted education globally, resulting in a move from in-person teaching to online teaching when educational institutions were forced to close (Yates et al., 2020). Moreover, increasing online activities among students is related to negative pedagogical impacts (Kandasamy et al., 2019; Geng et al., 2018). Over the past 25 years, concerns regarding PIU among young individuals have been raised (Lam et al., 2009; Simsek et al., 2019). PIU at its most extreme is considered by some to be a behavioral addiction (i.e., an addiction that does not involve the ingestion of a psychoactive substance) (Griffiths, 2005). However, since behavioral addictions may differ from drug addictions (Hellman et al., 2013), the present authors use the term ‘PIU’ rather than ‘internet addiction’ especially as leading scholars in the area have asserted that addictions *on* the internet (e.g., online gambling addiction) should not be considered as addictions *to* the internet (Griffiths, 2000) and that individuals are no more addicted to the internet than alcoholics are addicted to bottles (Gámez-Guadix et al., 2015; Griffiths & Pontes, 2014). PIU is defined as the excessive desire for internet use and comprises poor control over behaviors related to internet use, and is associated with distress and clinical impairment in daily activities (Li et al., 2019; Shaw & Black, 2008). Moreover, some argue that if ‘internet addiction’ and/or ‘PIU’ exist, it influences a relatively small percentage of online users and that what individuals on the internet are addicted to remains unclear (Widyanto et al., 2006). Therefore, PIU does not currently have an official diagnosis in diagnostic manuals, but studies have shown that PIU is related to psychological distress, which intensified during the COVID-19 outbreak and can lead to problems in academic, psychological, social, and occupational performance (Chen et al., 2022; Davis, 2001; Geng et al., 2018; Liang et al., 2022; Priego-Parra et al., 2020; Young & Rogers, 1998; Laconi et al., 2014; Young, 2016).

The risk of developing behavioral addictions, especially PIU, appears to be higher during adolescence and emerging adulthood compared with other cohorts (Grant et al., 2010; Przepiorka et al., 2019), and students appear to be one of the most affected demographic groups (Geng et al., 2018; Kandell, 1998) especially during the pandemic (Lin, 2020; Souza, 2021). However, a recent review of the prevalence of PIU during the pandemic reported there was no clear empirical evidence that it had increased during the pandemic (Burkauskas et al., 2022). Some studies have shown that in many countries, students spend more than two hours a day using the internet (Cotten & Jelenewicz, 2006; Crearie, 2014; Jones et al., 2009; Lee et al., 2017; Peng et al., 2019). If the internet is used to excess, it can have detrimental effects among a minority (Geng et al., 2018). Impacted individuals have problems with their academic performance and daily routines in comparison to students who are not impacted by their internet use (Chou & Hsiao, 2000; Kandasamy et al., 2019). Consequently, internet overuse has been associated with procrastination, which itself is associated with pedagogic problems (Geng et al., 2018; Kandemir, 2014a, b; Raiisi et al., 2018; Steel, 2007; Zhang et al., 2015).

Procrastination is defined as the deliberate delay in an action, despite being aware of its negative consequences (Klingsieck, 2013; Müller et al., 2020; Steel, 2007; Mortazavi et al., 2015; O’Brien, 2002; Van Eerde, 2003). Academic procrastination is highly prevalent among students (Schouwenburg et al., 2004; Solomon & Rothblum, 1984; Mohammadi Bytamar et al., 2017; Motie et al., 2012). In academic settings, it is very common for students to procrastinate when faced with assignments (Geng et al., 2018; Klassen et al., 2009; Lay & Silverman, 1996). One study reported that the prevalence of procrastination levels ranged from 29.2% to 47.9% among Iranian students and was similarly high in academic settings of other countries (Hayat et al., 2020a, b; Mahasneh et al., 2016; Özer et al., 2009). Therefore, procrastination should be taken seriously because of its prevalence among students (Onwuegbuzie, 2004).

Numerous studies have shown that procrastination is related to poor academic performance and low grades (Özer et al., 2009), anxiety, low self-esteem, and more generally, poor mental health (Steel, 2007). Several studies have examined the relationship between PIU and procrastination. Some studies have presented an association between PIU (including problematic social media and smartphone use) and procrastination (Hernández et al., 2019; Kandemir, 2014a, b; Lin, 2020; Przepiorka et al., 2016; Rozgonjuk et al., 2018; Şahin, 2014; Souza, 2021; Ozgonjuk et al., 2018). However, other studies have reported no significant relationship between PIU and procrastination (Odaci, 2011; Schraw et al., 2007). Therefore, the relationship between PIU and procrastination remains unclear. Despite extensive research examining PIU in Europe and the United States, little research has been done in Asia (Kljajic & Gaudreau, 2018), especially in Iran. Therefore, the present study aimed to examine the structural relationship between PIU and procrastination, while considering mediating variables.

Compensatory internet use theory (Kardefelt-Winther, 2014a, b) is relevant to the present study. Compensatory internet use theory proposes that life stressors motivate some individuals to excessively use the internet as a coping strategy to help deal with their negative emotions. A number of studies have shown empirical support for compensatory internet use theory in explaining problematic smartphone use (Elhai & Contractor, 2018; Elhai et al., 2018a, b; Long et al., 2016). Compensatory internet use theory also suggests individuals with high tolerance for ambiguity (defined below) have less motivation to engage in internet use (Elhai et al., 2018a, b).

When students face a task in academic settings, they often resort to procrastination and delay the task until the last moment (Geng et al., 2018). In contrast to unpleasant academic tasks, quick access to the rewarding content in cyberspace and social networks seems to be a welcome escape for students. Coping with media exposure is one of the most important sources of goal conflict in everyday life (Reinecke & Hofmann, 2016). Moreover, engaging in online media use is actively used to delay homework and other commitments (Hinsch & Sheldon, 2013; Meier et al., 2016).

Negative emotions have been suggested as one of the antecedents of procrastination (Steel, 2007; Tice et al., 2001; Wohl et al., 2010). Evidence has shown that individuals engage in more procrastination when they are upset or sad, and that distraction reduces the negative feelings of procrastination (Tice et al., 2001). Moreover, depressed mood, neuroticism, and distressing situations have been associated with procrastination (Kınık et al., 2020; Moslemi et al., 2020). Therefore, emotion regulation plays an important role in procrastination (Sirois et al., 2019; Tice & Bratslavsky, 2000).

According to Gross et al. (2006), emotion regulation is a subset of affect regulation. Emotion regulation refers to how individuals intensify or prevent their emotions from occurring according to their goals (John & Gross, 2007; Balzarotti et al., 2010; Gross, 1998; Melka et al., 2011). The dimensions of the concept of emotion regulation comprise: (i) awareness and understanding of emotions; (ii) accepting emotions; (iii) the ability to control impulsive behaviors when experiencing negative emotions; and (iv) the ability to flexibly use emotional adjustment strategies tailored to the situation to regulate emotional responses, achieve individual goals, and meet situational demands (Amendola et al., 2019).

At the broadest level, according to Gross and John's theory of emotion regulation (2003), the present study distinguishes between antecedent-focused and response-focused emotion regulation strategies. An antecedent-focused strategy is something an individual does before their emotion response tendencies fully activate and change their behavior and peripheral physiological responses. In response-focused strategies, individuals take actions after an emotion has already begun and response tendencies have already developed. A smaller number of well-defined strategies have been studied rather than all of the many emotion regulation strategies at once. In selecting strategies for study, a number of factors were considered. The first requirement is that the methods should be ones that individuals use on a regular basis and the second requirement was that one example of each antecedent-focused and response-focused strategies should be included. A cognitive reappraisal and an expressive suppression strategy met these criteria (Gross et al., 2003). Emotion regulation strategies are categorized into two broad classes: cognitive reappraisal (an antecedent-focused strategy) and expressive suppression (a response-focused strategy) (Gross & John, 1998; John & Gross, 2007). Reappraisal is defined as changing a potentially emotional eliciting situation to reduce the emotional impact of the situation (Gross & John, 2003).

Evidence suggests that individuals who typically regulate their emotional experiences through the reappraisal strategy report more positive and fewer negative emotions. Also, they experience higher levels of psychological well-being (John & Gross, 2007). However, individuals who manage their emotions using the suppression strategy report more negative emotions, fewer positive emotions, less social support, and more depression (John & Gross, 2007; Gross & Jazaieri, 2014; Heatherton & Tice, 1994). A study in China reported that there is a relationship between individual affect, relationship satisfaction, and PIU (Zeng et al., 2021). During the pandemic, another Chinese study by Liang et al. (2021) reported that cognitive reappraisal and expression suppression were related to problematic internet use among adolescents (Liang et al., 2021). Notably, problematic users of social networking sites experience more problems with emotion regulation than non-problematic users (Hormes et al., 2014; Spada & Marino, 2017). Procrastination can be viewed as a failure of emotion regulation, which results in an individual’s desire to temporarily feel good (Sirois & Pychyl, 2013; Wypych et al., 2018). Procrastination may result from negative emotions, such as fear of failure (Haghbin et al., 2012; Schouwenburg, 1992; Schraw et al., 2007) or discomfort intolerance (Harrington, 2005). According to Blunt and Pychyl (2000), negative emotions caused by tasks are related to the avoidance of such tasks. Overall, poor emotion regulation strategies are related to procrastination via two mechanisms. First, as noted by Tice and Bratslavsky (2000), low emotion regulation skills reduce self-control, and poor self-control is associated with procrastination (Kim et al., 2017). Second, delaying assignments or tasks can be a strategy for short-term mood enhancement.

As aforementioned, the use of media and the internet can serve as a means for procrastination behaviors to temporarily reduce negative emotions and change mood in the face of difficult tasks (Reinecke & Hofmann, 2016; Zillmann, 1988). Research has shown a significant relationship between internet use and emotion regulation (Caplan, 2002, 2010; Faghani et al., 2020; Liang et al., 2021), and suggests that mood regulation can motivate many online activities (Bessière et al., 2004; LaRose et al., 2003). Based on the theory of compensatory internet use (Kardefelt-Winther, 2014a, b), internet use can be considered as a coping strategy to deal with negative emotions (Evren et al., 2019; Young, 1998).

Tolerance for ambiguity has drawn attention in recent years because of its transdiagnostic role in many disorders and mental health problems, such as anxiety disorders, obsessive–compulsive disorder, and mood disorders. It is defined as a behavioral, cognitive, and negative emotional response to uncertainty in vague situations (Carleton, 2016; Budner, 1962; MacDonald, 1970). Tolerance for ambiguity is closely related to the intolerance of emotional distress and negative emotions (Leyro et al., 2010), and is based on the theory of compensatory internet use. Internet use can be considered a coping strategy to deal with such emotions (Faghani et al., 2020). Uncertainty intolerance is associated with internet use (Elhai et al., 2018a, b). Several studies have suggested that tolerance for ambiguity is related to problematic smartphone use (Carleton et al., 2019; He et al., 2021; Rozgonjuk, et al., 2019). A recent study among Chinese college students indicated that tolerance for ambiguity is directly and indirectly (via mediators) associated with academic procrastination (Xu & Hu, 2018). Moreover, uncertainty paralysis (i.e., a tendency to freeze during uncertainty), as one of the facets of tolerance for ambiguity, can be considered a reflection of a procrastination response to vague tasks (Haycock et al., 1998). Also, Doğanülkü et al. (2021) reported that fear of COVID-19 and intolerance of uncertainty were related to procrastination among Turkish students.

### Present study hypotheses

In the review of the literature, two types of relationships have been observed regarding PIU and procrastination. Some studies have suggested procrastination as a mediator of PIU (Cui et al., 2021; Geng et al., 2018; Gong et al., 2021; Hayat et al., 2020a, b; Kandemir, 2014a, b; Thatcher et al., 2008), while others have indicated the reverse (Hernández et al., 2019; Hong et al., 2021; Reinecke et al., 2018), suggesting the direction of the relationship between PIU and procrastination is still unclear. Therefore, the first hypothesis of the present study was that there would be a direct relationship between PIU and procrastination (H_1_). Instead of focusing on academic goals, students may seek a source of immediate reward in internet use, and may eventually become distracted from their academic activities (Kandemir, 2014a, b). To carefully examine the relationship between PIU and procrastination, three variables (tolerance for ambiguity, suppression, and reappraisal) were examined as mediators. Therefore, the second hypothesis was that tolerance for ambiguity would mediate the relationship between PIU and procrastination (H_2_).

The problematic use of the internet, as a coping strategy to deal with anxiety, is related with uncertainty (Spada et al., 2008). Therefore, the third hypothesis was that reappraisal would mediate the relationship between PIU and procrastination (H_3_). Individuals with higher reappraisal as a desirable emotion regulation strategy experience less procrastination (Sirois et al., 2019). Moreover, the fourth hypothesis was that suppression would mediate the relationship between PIU and procrastination (H_4_). It is suggested that individuals turn to procrastination strategies to avoid negative emotions and experience better feelings (Baumeister et al., 1994).

### Aims

To the best of the authors’ knowledge, the present study is the first to study the relationship between PIU and procrastination via mediating roles of tolerance for ambiguity, reappraisal, and suppression among Iranian students. The present study analyzes two different directions of relationship between PIU and procrastination to assess which model best fits the data: (i) PIU as input and procrastination as output, and (ii) procrastination as input and PIU as output. Moreover, the study investigated whether age and gender influenced the relationship between PIU and procrastination.

## Methods

### Participants and procedure

The present study was approved by the Research Ethics Committee of Kharazmi University. Participants were recruited from four universities in the cities of Tehran and Karaj (Kharazmi University, Tehran University, Shahid Beheshti University, and Sharif University of Technology). The final sample comprised 434 students with 206 males (mean age = 24.43 years, *SD* = 2.88 years; age range: 18–30 years) and 228 female students (mean age = 23.15 years, *SD* = 2.85 years; age range: 18–28 years). Overall, 30%, 28%, 27%, and 15% of the participants were studying in the faculties of Engineering, Humanities, Natural Sciences, and Mathematics, respectively. A survey including questions concerning basic demographics, procrastination, PIU, tolerance for ambiguity, reappraisal, and suppression was completed by each participant. The survey was completed offline and took approximately 30 min to complete. Before starting the survey, participants were informed about the study’s aims, and provided written informed consent. The survey’s questions were rotated in order to control order effects. Participants were assured that their information would be kept confidential and notified that they could withdraw their data before data analysis. A total of 500 surveys were distributed and 480 were returned.

### Measures

#### Internet Addiction Test (IAT)

The 20-item IAT (Young, 1998; Young et al., 1998; Persian version: (Alavi et al., 2011) was used to assess problematic internet use. The items (e.g., “*I tried to hide the time I spent on the internet from others*”) are rated on a five-point scale, from 1 (*not applicable*) to 5 (*always*). Higher scores indicate a greater risk of PIU. Confirmatory Factor Analysis (CFA) showed that the Persian IAT fitted the data well (χ^2^/df = 1.58, CFI = 0.95, TLI = 0.96, RMSEA = 0.04, and SRMR = 0.03) (Appendix Figs. 2 and 3). The internal consistency in the present study was excellent (alpha = 0.90).

#### Academic Procrastination Scale (APS)

The 27-item APS (Solomon & Rothblum, 1984; Persian version: Jokar & Delavarpour, 2007) was used to assess academic procrastination. The items (e.g., “*To what degree do you procrastinate on this task*?”) are rated on a five-point scale, ranging from 1 (*never*) to 5 (*always*). Higher scores indicate greater procrastination. The CFA showed that the Persian APS fitted the data well (χ^2^/df = 2.04, CFI = 0.96, TLI = 0.94, RMSEA = 0.05, and SRMR = 0.04) (Appendix Figs. 4 and 5). The internal consistency in the present study was very good (alpha = 0.82).

#### Multiple Stimulus Types Ambiguity Tolerance Test (MSTAT)

The 22-item MSTAT (McLain, 1993; Persian version: Abolqasemi & Narimani, 2005) was used to assess tolerance for ambiguity (TFA). The items (e.g., “*I don’t tolerate ambiguous situations well*”) are rated on a five-point scale ranging from 1 (*strongly disagree*) to 5 (*strongly agree*). Lower scores indicate greater tolerance for ambiguity. The CFA showed that the Persian MSTAT fitted the data well (χ^2^/df = 1.28, CFI = 0.96, TLI = 0.95, RMSEA = 0.04, and SRMR = 0.04) (Appendix Figs. 6 and 7). The internal consistency in the present study was very good (alpha = 0.88).

#### Emotion Regulation Questionnaire (ERQ)

The 10-item ERQ (Gross & John, 2003; Persian version: Hasani, 2016) was used to assess emotion regulation. The scale comprises two dimensions: habitual expressive suppression (with four items; e.g., *“I keep my emotions to myself”*) and cognitive reappraisal (with six items; e.g., *“When I’m faced with a stressful situation, I make myself think about it in a way that helps me stay calm”*). Items are rated on a seven-point scale ranging from 1 (*strongly disagree*) to 7 (*strongly agree*). The CFA showed that the Persian ERQ fitted the data well (χ^2^/df = 1.49, CFI = 0.96, TLI = 0.95, RMSEA = 0.04, and SRMR = 0.03) (Appendix Figs. 8 and 9). The internal consistencies for the subscales of the ERQ were very good in the present study (reappraisal α = 0.88 and suppression α = 0.84).

### Design and data analysis

In the present study, structural equation modeling (Martinussen, 2010) was used to explore the structural relationships between PIU, TFA, reappraisal, suppression, and procrastination within a conceptual model. In order to identify the best model of reciprocal relationship between PIU and procrastination, the study investigated the conceptual model in two formats, considering the moderator variables. In the first model, PIU was considered as endogenous and procrastination as exogenous variable, while in the second model procrastination was taken as the endogenous variable and PIU as the exogenous variable. It should be noted that the best fit model was considered the fundamental model. In addition, the study examined whether the best-fitting model exhibited measurement invariance based on sex and age groups (18 to 24 years old and 25 to 30 years old). Commonly, measurement invariance is considered as acceptable when the changes in CFI (ΔCFI) are less than 0.01 (Cheung & Rensvold, 2002).

To do this, the data were analyzed with SPSS-24 and AMOS-24 software. Before the analyses, the assumptions of structural equation modeling were checked and obtained. Since the pattern of the missing values of the obtained data was random, cases with missing values of more than 5% were removed (Tabachnick et al., 2007; Davey & Salve, 2010). Out of 480 participants, the data of 35 individuals were detected with missing values of more than 5% of the total endorsement. Therefore, data from 445 participants were used in the analyses. Next, independent samples *t-*tests were used to test the extent to which there was a relationship between the missing values and the investigated variables (Davey & Savla, 2010; Ho, 2014). The results showed no systematic relationship between the missing values and other variables. Therefore, using regression-based imputation, the missing observations were replaced with a predicted score generated by multiple regression on the non-missing scores of other variables (Davey & Savla, 2010; Ho, 2014). Moreover, box plots were developed to ensure that all data remained within the first and fourth quartile boundaries. In addition, the outliers were removed using Mahalanobis distance (Meyers, et al., 2006). Because of the outlier analysis, 11 cases were treated as outliers and excluded from the final analyses. Finally, data from 434 participants were used in the final analysis.

Initially, the correlations between the research variables were calculated. To examine the theoretical model, the recommended two-step process of Anderson and Gerbing (1988) was applied. Accordingly, the reliability and validity of the present study measures were first verified using the CFA, and then, the theoretical model using structural equation modeling in AMOS software was established. A maximum likelihood estimator was used in the assumed model, and estimated the fit of the model at two levels.

In the first level, to assess model fit, more popular fitness indices, including chi-square with its degrees of freedom, comparative fit index (CFI), goodness of fit index (GFI), non-normed fit index (NNFI), standardized root mean square residual (SRMR), and the root mean square error of approximation (RMSEA) were used. Usually, the fit is considered good when χ^2^/*df* < 3, CFI > 0.90, GFI > 0.90, NNFI > 0.90, SRMR < 0.08, and RMSEA < 0.06 (see Hu & Bentler, 1999; Meyers et al., 2006).

In the second level, the coefficients of the model’s paths and the coefficients of determination of endogenous variables were investigated. Bootstrap analysis (iteration number = 500) was used to test the mediation model’s indirect effects’ significance level. Furthermore, exploratory factor analyses for PIU, TFA, procrastination, suppression, and reappraisal variables (based on Matsunaga's (2008) solution for item parceling one-dimensional scales) were performed. The analysis was conducted with three fixed factors and varimax rotation to select the marker variables. PIU, TFA, procrastination, suppression, and reappraisal were considered as marker variables, and the CFA assessed their unidimensionality. All the parcels were chosen as observable variables because the factor loadings were above 0.30 (see Kline, 2015). Finally, the three parcels of each variable were considered as markers (Table 1).

**Table 1:** Items and factor loadings for parcels of the variables

Variables	Parcel 1		Parcel 2		Parcel 3	
	Item	Factor loading	Item	Factor loading	Item	Factor loading
Procrastination	12	0.69	25	0.65	18	0.77
	9	0.65	21	0.64	19	0.77
	24	0.65	6	0.63	27	0.73
	20	0.63	23	0.62	8	0.73
	17	0.63	16	0.61	26	0.72
	10	0.60	4	0.58	7	0.45
	5	0.59	11	0.57		
	14	0.57	2	0.55		
	1	0.55	15	0.50		
	22	0.52	13	0.48		
	3	0.49				
PIU	19	0.91	2	0.75	12	0.93
	20	0.80	1	0.76	14	0.77
	18	0.79	6	0.60	10	0.66
	16	0.65	9	0.64	15	0.58
	13	0.67	8	0.52	11	0.52
	3	0.50	7	0.59		
	17	0.51	4	0.54		
			5	0.44		
Tolerance for ambiguity	6	0.73	16	0.76	14	0.76
	9	0.70	22	0.76	15	0.76
	10	0.70	12	0.70	4	0.54
	5	0.69	21	0.68	20	0.53
	1	0.69	11	0.65	3	0.50
	13	0.68	19	0.59		
	18	0.68	17	0.55		
	2	0.65	7	0.52		
	8	0.53				
Reappraisal	10	0.83	7	0.89	5	0.92
	3	0.76	8	0.68		
	1	0.71				
Suppression	4	0.89	9	0.94	6	0.91
	2	0.77				

## Results

### Descriptive analyses

Table 2 presents demographic information of the study sample.

**Table 2:** Demographic variables of the study sample

Variable	Group	Frequency	%
Gender	Female	228	52.5
	Male	206	47.5
Academic attainment	Undergraduate	165	38
	Postgraduate	269	62
Marital status	Single	390	89.9
	Married	44	10.1
Internet use time per day (h)	1–2	22	5.1
	3–5	370	85.3
	> 5	42	9.7

### Initial analysis

Table 3 reports the kurtosis, skewness, means, standard divisions, and zero-order correlations of the present study’s variables. As shown in Table 3, lack of multicollinearity (*r* < 0.85) was confirmed between the variables (Kline, 2011). The prerequisite for normal distribution of kurtosis was confirmed (range − 0.34 to 1.03) as well as the variables’ skewness values (range − 0.97 to 0.55) (Hu & Bentler, 1999; Kline, 2011). Results showed that PIU and TFA were positively correlated with procrastination, and the other mediating variables were significantly associated with PIU, other than for suppression and reappraisal. The three mediators were significantly associated with the exogenous variables. Moreover, an adequate fit of the measurement model (χ^2^ = 183.63, *p* < 0.001, df = 80; χ^2^/df = 2.29, CFI = 0.96, NFI = 0.99, RMSEA = 0.05, GFI = 0.95, SRMR = 0.047, IFI = 0.96) was confirmed by the fit indices of a CFA. Table 4 shows the selected markers of the variables.

**Table 3:** Descriptive statistics and the zero-order correlations of the research variables

Variables	M	SD	Skewness	Kurtosis	1	2	3	4	5
1. PRO	73.64	15.72	-0.97	1.03	1				
2. PIU	40.19	11.78	0.55	-0.33	0.29***	1			
3. TFA	66.26	10.17	-0.32	0.79	0.34***	0.21***	1		
4. REAP	26.13	8.40	-0.12	-0.34	-0.34***	-0.25***	-0.28***	1	
5. SUP	16.45	6.98	0.51	0.30	0.32***	-0.23***	0.15***	-0.07	1

*M* Mean; *SD* Standard division; *PRO* Procrastination; *PIU* Problematic internet use; *TFA* Tolerance for ambiguity; *REAP* Reappraisal; *SUP* Suppression. Multicollinearity (*r<*0.85) was not found, according to Kline (2015). *p<.05. **p<.01. ***p<.001. (*N=*434)

**Table 4:** Non-standardized coefficients, the observable variables’ *t*-values in the measurement model, and standardized coefficients

Variables	Non-standardized Coefficients	Standardized coefficients	*t*
Procrastination			
Pro1	3.54	0.74	14.21
Pro2	2.49	0.69	13.44
Pro3	1.09	0.56	10.75
PIU			
PIU1	3.90	0.76	15.90
PIU2	3.17	0.69	14.28
PIU3	3.30	0.73	15.10
Tolerance for ambiguity			
TFA1	3.21	0.69	13.39
TFA2	3.11	0.72	14.08
TFA3	2.33	0.65	12.30
Reappraisal			
REAP1	3.09	0.68	11.21
REAP2	1.24	0.49	8.55
REAP3	1.17	0.57	9.72
Suppression			
SUP1	3.93	0.59	11.79
SUP2	2.49	0.76	15.96
SUP3	1.09	0.72	15.01

*M* Mean; *SD* Standard division; *PRO* Procrastination; *PIU* Problematic internet use; *TFA* Tolerance for ambiguity; *REAP* Reappraisal; *SUP* Suppression. Multicollinearity (*r<*0.85) was not found, according to Kline (2015). *p<.05. **p<.01. ***p<.001. (*N=*434)

### Testing the mediated associations

As forementioned, the study investigated whether Model 1 (PIU as endogenous and procrastination as endogenous variable) or Model 2 (procrastination as the endogenous variable and the PIU as the exogenous variable) provided the best fit for the data. The fit statistics showed that the Model 1 (χ^2^ = 207.27, *p* < 0.001, df = 80; χ^2^/df = 2.50, CFI = 0.95, NFI = 0.96, RMSEA = 0.05, GFI = 0.97, SRMR = 0.04, IFI = 0.97) indicated a good fit of the research model, whereas fit indices were weak for Model 2 (χ^2^ = 191.49, *p* < 0.001, df = 83; χ^2^/df = 2.31, CFI = 0.85, NFI = 0.81, RMSEA = 0.12, GFI = 0.67, SRMR = 0.34, IFI = 0.68). Therefore, the fit indices of the data in the two models indicated that the PIU as the endogenous variable fitted better than as exogenous variable. Therefore, mediated associations were carried out with respect to the PIU as endogenous.

**Fig. 1:**
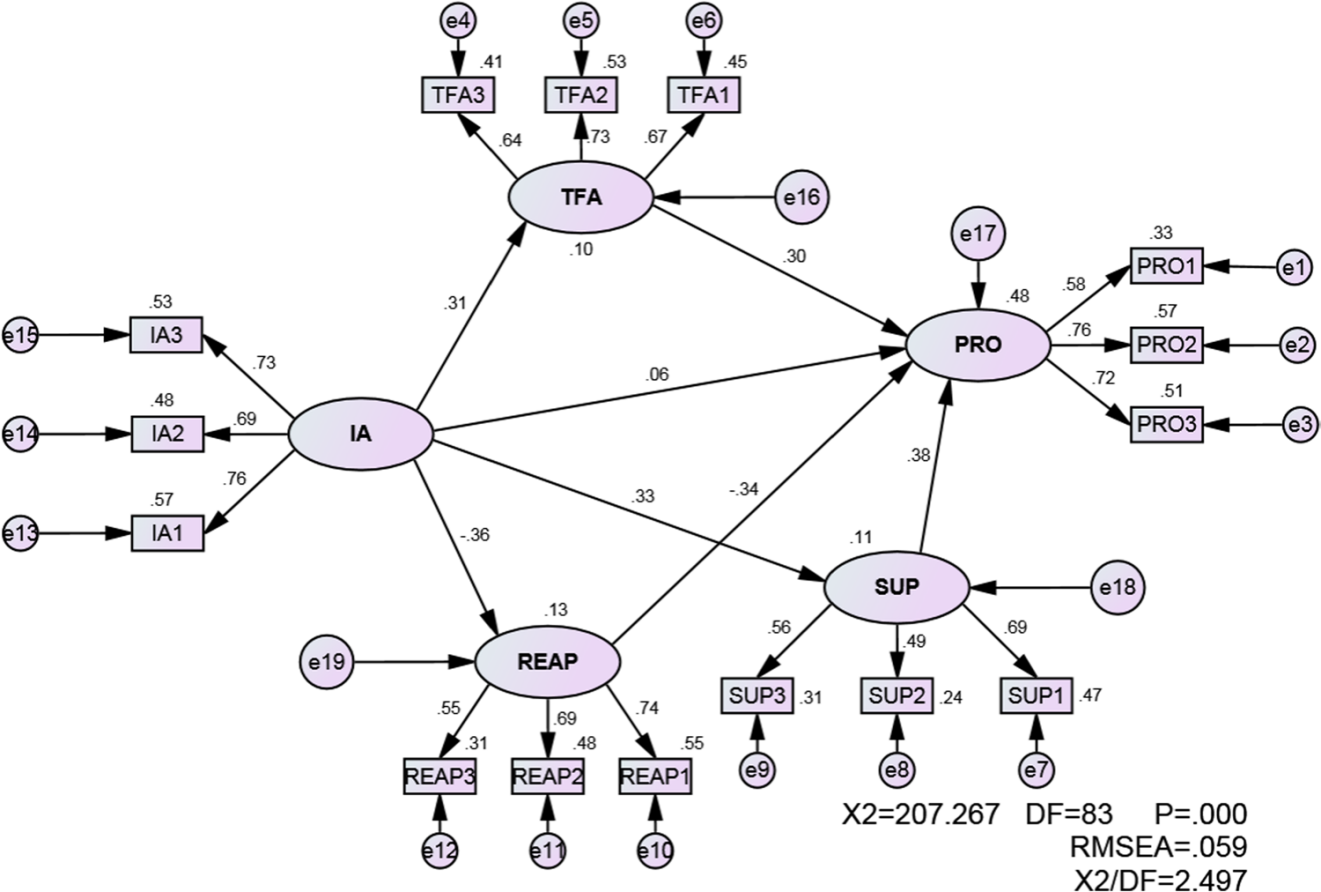
Final results of the research model

As expected, a significant χ^2^ value was obtained because it is easily affected by sample size, and often appears significant in large samples (Meyers et al., 2006).The current model explained 48% of the variance in procrastination scores. As shown in Table 5 and Fig. 1, the direct relationship between PIU and procrastination (β = 0.06, *t* = 0.84) was not significant, while tolerance for ambiguity (β = 0.40, *t* = 4.43), reappraisal (β = -0.34, *t* = -4.83), and suppression (β = 0.38, *t* = 4.66) predicted procrastination. Also, PIU significantly predicted tolerance for ambiguity (β = 0.31, *t* = 4.58), reappraisal (β = -0.36, *t* = -5.33), and suppression (β = 0.33, *t* = 4.64).

**Table 5:** Direct effects of the latent variables

Independent variables	Dependent variables	Β	T	SE	*p*	R^2^
PIU	TFA	0.31	4.58	0.041	0.001	0.10
PIU	REAP	-0.36	-5.33	0.062	0.001	0.13
PIU	SUP	0.33	4.64	0.058	0.001	0.11
PIU	PRO	0.06	0.84	0.071	0.42	
TFA	PRO	0.40	4.43	0.111	0.001	
REAP	PRO	-0.34	-4.83	0.077	0.001	
SUP	PRO	0.38	4.66	0.100	0.001	

*PRO* Procrastination; *PIU* Problematic internet use; *TFA* Tolerance for ambiguity; *REAP* Reappraisal; *SUP* Suppression

### Bootstrapping

Bootstrap analysis (iteration number = 500) was performed to evaluate the mediation model. As shown in Table 6, there was a notable pathway from PIU to procrastination via TFA (β = 0.093; SE = 0.012; 95% CI ranges from 0.037 to 0.135; *p* < 0.05), reappraisal (β = -0.122; SE = 0.025; 95% CI ranges from -0.185 to -0.085, *p* < 0.05), and suppression (β = 0.125; SE = 0.018; 95% CI ranges from 0.110 to 0.129, *p* < 0.05). In total, the results indicated a full mediation model.

**Table 6:** Bootstrapping indirect effect with 95% confidence interval for the mediation model

Independent variable	Mediating variable	Dependent variable	Standard coefficient	Standard error	95% CI		*p*
					Lower	Upper	
PIU	TFA	PRO	0.093	0.012	0.037	0.135	0.001
PIU	REAP	PRO	-0.122	0.025	-0.185	-0.085	0.001
PIU	SUP	PRO	0.125	0.018	0.110	0.129	0.001

*PRO* Procrastination; *PIU* Problematic internet use; *TFA* Tolerance for ambiguity; *REAP* Reappraisal; *SUP* Suppression

### Measurement invariance of final model in sex and age groups

In order to examine the role of sex and age groups, the final model was used to assess measurement invariance (i.e., to examine the equivalence of this model across gender and age groups). The results are shown in Table 6. The change in CFI (ΔCFI) was selected as the comparative index. As shown in Table 6, the measurement invariance analyses demonstrated that invariance of final model across gender and across age group was tenable, with all ΔCFI < 0.01. In addition, all the other model fit indices (IFI, TLI, CFI, SRMR, RMSEA) met the criterion. In sum, the final model showed desirable measurement invariance based on gender and age groups (Table 7).

**Table 7:** Fit indices for the measurement invariance of final model according to sex and age groups

		χ^2^	DF	IFI	TLI	CFI	SRMR	ΔCFI	RMSEA (90%CI)
Across sex	Girl (n = 228)	121.35	58	0.95	0.94	0.95	0.04	< 0.01	0.045 (0.042, 0.048)
	Boy (n = 206)	128.35	53	0.96	0.95	0.96	0.05	< 0.01	0.039 (0.036, 0.042)
Across age	18 to 24 YO (n = 227)	136.38	58	0.94	0.93	0.95	0.06	< 0.01	0.042 (0.038, 0.046)
	25 t0 30 YO (n = 207)	141.48	53	0.93	0.92	0.93	0.05	< 0.01	0.035 (0.032, 0.039)

*RMSEA* Root mean square error of approximation; *CI* Confidence interval; *CFI* Comparative fit index; *TFI* Tucker–Lewis index; *ΔCFI* Change in CFI; *DF* Degrees of freedom

## Discussion

The present study investigated the relationship between PIU and procrastination, which was examined with two specific objectives. First, the direct relationship between PIU and procrastination was examined, and then, their indirect relationship with the mediation of three variables (tolerance for ambiguity, reappraisal, and suppression) was examined. Four hypotheses were tested and the results indicated there was: (i) a direct relationship between PIU and procrastination; (ii) an indirect relationship between PIU and procrastination, mediated by IU; and (iii) an indirect relationship between PIU and procrastination mediated by reappraisal and suppression.

Two different directions of relationship between PIU and procrastination were analyzed to assess which model best fitted the data: (i) PIU as input and procrastination as output, and (ii) procrastination as input and PIU as output. The analysis showed that the first model was a better fit for the data. Research has shown that PIU is related to procrastination (Kandemir, 2014a, b; Thatcher et al., 2008). The results of the present study showed no direct relationship between PIU and procrastination (H_1_) because of the predictive power of the mediators. According to Baron and Kenny (1986) and Sobel (1982), the mediators can explain a part of or the entire relationship between two variables. Accordingly, the findings confirmed H_2_, H_3_ and H_4_ as well as the mediation model. However, considering the fact that because the whole relationship between PIU and procrastination was mediated by other variables, another interpretation is that if the mediators were controlled for, the relationship disappeared and it is possible that the role of PIU as a predictor of procrastination was non-significant. Notably, the majority of previous studies have considered procrastination as a predictor of PIU (e.g., Hernández et al., 2019; Şahin, 2014). However, the present results indicated the possibility of predominance of PIU over procrastination via an indirect relationship.

According to H_2_, the relationship between PIU and procrastination would be mediated by tolerance for ambiguity. High levels of social anxiety are associated with high levels of tolerance for ambiguity (Boelen & Reijntjes, 2009; Carleton, 2012; Carleton et al., 2010) and social avoidance (Miers et al., 2014). Therefore, tolerance for ambiguity is associated with an individual’s preference for online social interaction. Also, social anxiety has been associated with problematic smartphone use (Wolniewicz et al., 2018) and non-social smartphone use (Elhai et al., 2017).

The findings of the present study also support the relationship between tolerance for ambiguity and procrastination. Faced with difficult and ambiguous assignments, students may experience uncertainty paralysis, a subset of tolerance for ambiguity, procrastinating instead of completing homework (Haycock et al., 1998). The findings also supported that suppression and reappraisal mediated the relationship between PIU and procrastination. Accordingly, self-regulatory executive function theory (Wells & Matthews, 1996) states that acute addictive behaviors are related to negative metacognitive beliefs concerning non-controllability, self-doubt, maladaptive coping strategies, and further poor metacognitive strategies (Spada, 2015), which eventually cause psychological and behavioral disorders. PIU is associated with negative emotions (Cheng & Li, 2014) among individuals who use procrastination to regulate their negative emotions to temporarily avoid negative emotions and experience better feelings (Baumeister et al., 1994) by increasing short-term pleasure and reducing short-term pain (Gross & Jazaieri, 2014; Tamir, 2009). Experiencing short-term pleasure is the motivation that relates to procrastination as a way to regulate dysfunctional emotions. The results of the present study also showed that reappraisal was inversely related to procrastination. Individuals who re-evaluate their stressors and problems reduce their negative emotions (Finlay-Jones, 2017), and reduce the likelihood of engaging in procrastination (Sirois et al., 2019).

The results showed measurement invariance of the current model across gender and age groups, indicating that there were no differences across ages and gender in the current model. Therefore, gender and age do not influence the relationship between PIU and procrastination. More broadly, the results of the present study provide some support for the compensatory internet use theory (Kardefelt-Winther, 2014a, b), which posits that some individuals regulate their negative emotions by using the internet problematically. Individuals who score high on tolerance for ambiguity spend many hours on the internet to reduce the severity of their ambiguity and anxiety (Spada et al., 2008). According to Young (1998), PIU can occur when individuals use the internet to cope with difficult life situations, such as exams and academic tasks. According to Young (1998), internet use has a propensity to be used to cope with or compensate for real life problems. In later works, Caplan and High (2011) also suggested that through the exchange of online messages, individuals compensate for what they may lack in real life. According to compensatory internet use theory, negative life situations can motivate internet users to spend time online to alleviate negative emotions (Kardefelt-Winther, 2014a, b). As a result of social stimulation, the individual feels better. It is an understandable and practical way to acquire social stimulation when there is a lack of it (e.g., Chappell et al., 2006), but this habit may sometimes be associated with negative consequences such as procrastination due to the amount of compensation required to regulate negative feelings.

### Limitations

The present study has several limitations. First, the study sample was selected from Iranian universities, and mainly included students living in dormitories. Therefore, the results of this research cannot be generalized to all students or to those from other countries. Second, from the perspective of the social-cognitive models of media use, it is difficult to distinguish between problematic and addictive use of the internet because of the widespread use of the internet and social networks. Therefore, spending many hours on the internet is not necessarily problematic (LaRose & Eastin, 2004; LaRose et al., 2010; Tokunaga, 2017). Third, data were collected using self-report instruments, potentially leading to biased or distorted responses. Fourth, some control variables such as mental health and life stress were not assessed in the present study. Fifth, the IAT was used to determine PIU which might not be an effective tool in comparison with other PIU scales (such as the IDS-15 developed by Pontes et al., 2017). However, the IAT is the psychometric scale most used in Iran. Finally, the cross-sectional design employed does not allow for definitive statements of causality. Furthermore, because of full mediation, if mediators were controlled, the relationship between PIU and procrastination disappeared, therefore, it is not clear if tolerance for ambiguity, reappraisal, and suppression are mediators or control variables. Longitudinal analyses might provide further insight on the issue, but it is impossible to definitively conclude that the results of the present study support the importance of the PIU construct given that it is limited by the cross-sectional nature of the data.

## Conclusion

The results of the present study expand on the findings of previous studies in explaining the relationship between PIU and procrastination, and explained the underlying constructs of students’ procrastination. Suppression, reappraisal, and tolerance for ambiguity mediated the relationship between PIU and procrastination, offering a more nuanced explanation of this relationship than previous research.

## Figures and Tables

**Fig. 2:**
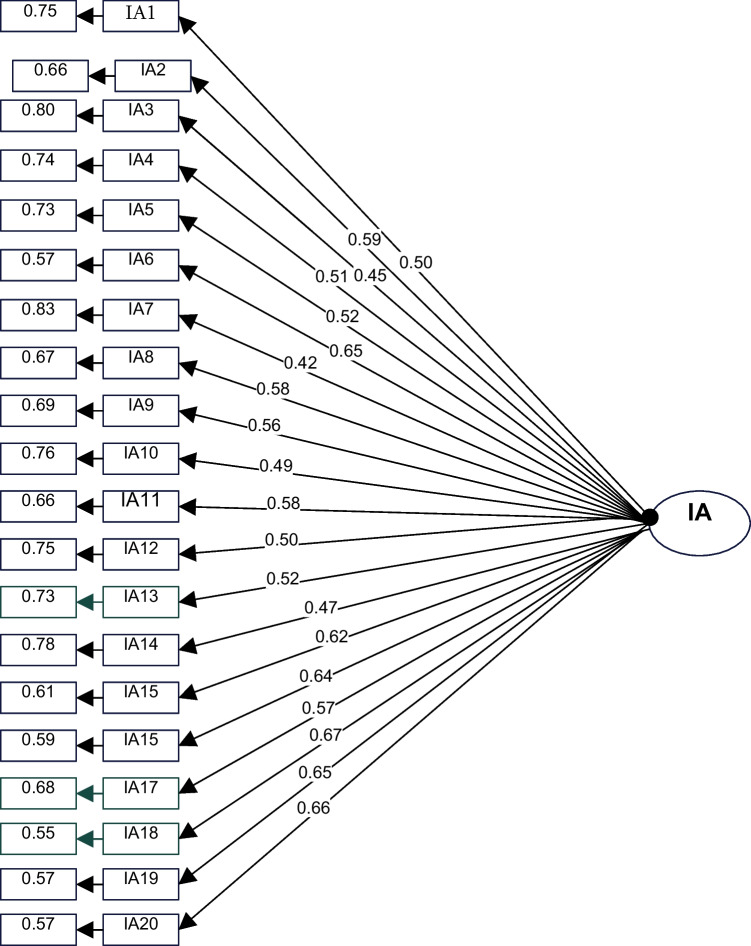
CFA for internet addiction scale (standard solution). Chi-square = 268.6, *df* = 170, *p*-value < 0.0001, RMSEA = 0.044

**Fig. 3:**
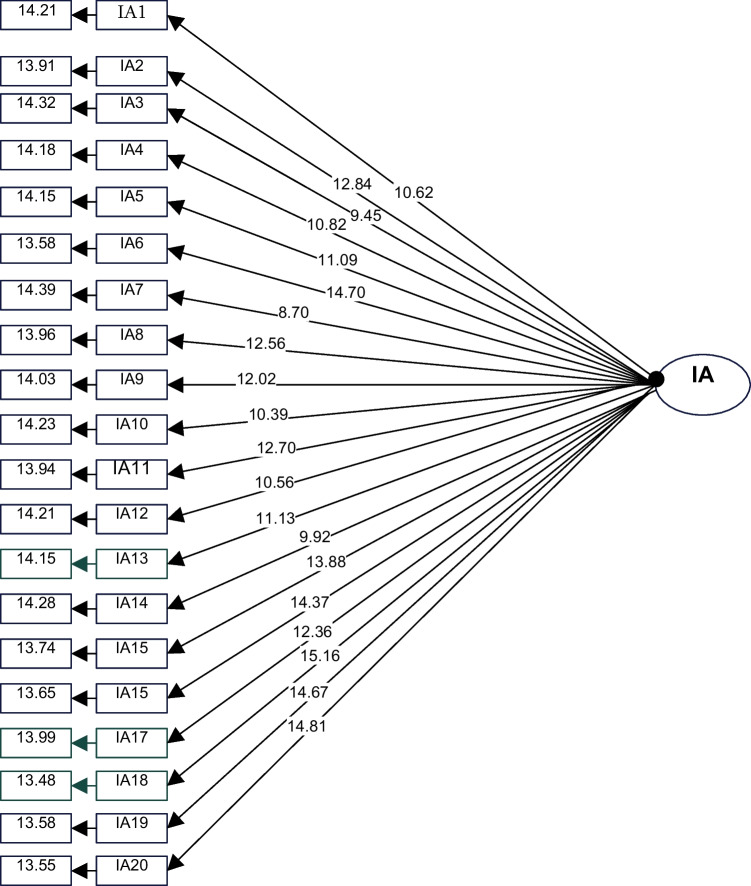
CFA for internet addiction scale (*t*-values). Chi-square = 268.6, *df* = 170, *p*-value < 0.0001, RMSEA = 0.044

**Fig. 4:**
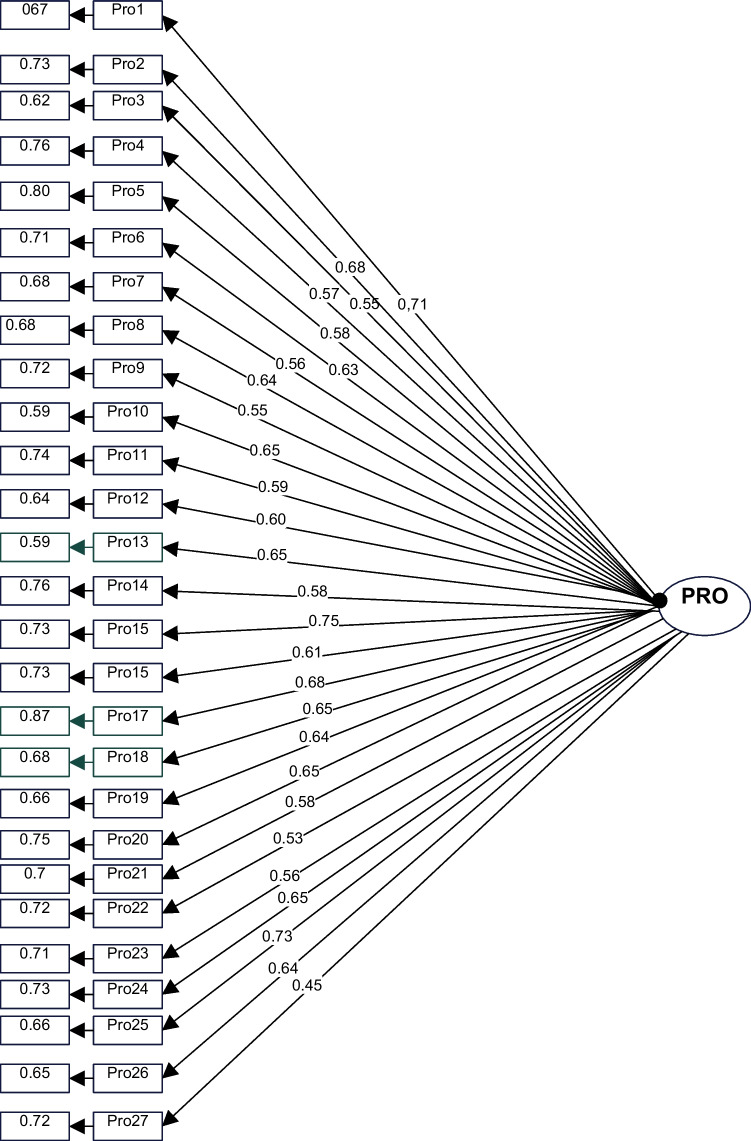
CFA for academic procrastination (standard solution). Chi-square = 660.96, *df* = 324, *p*-value < 0.0001, RMSEA = 0.053

**Fig. 5:**
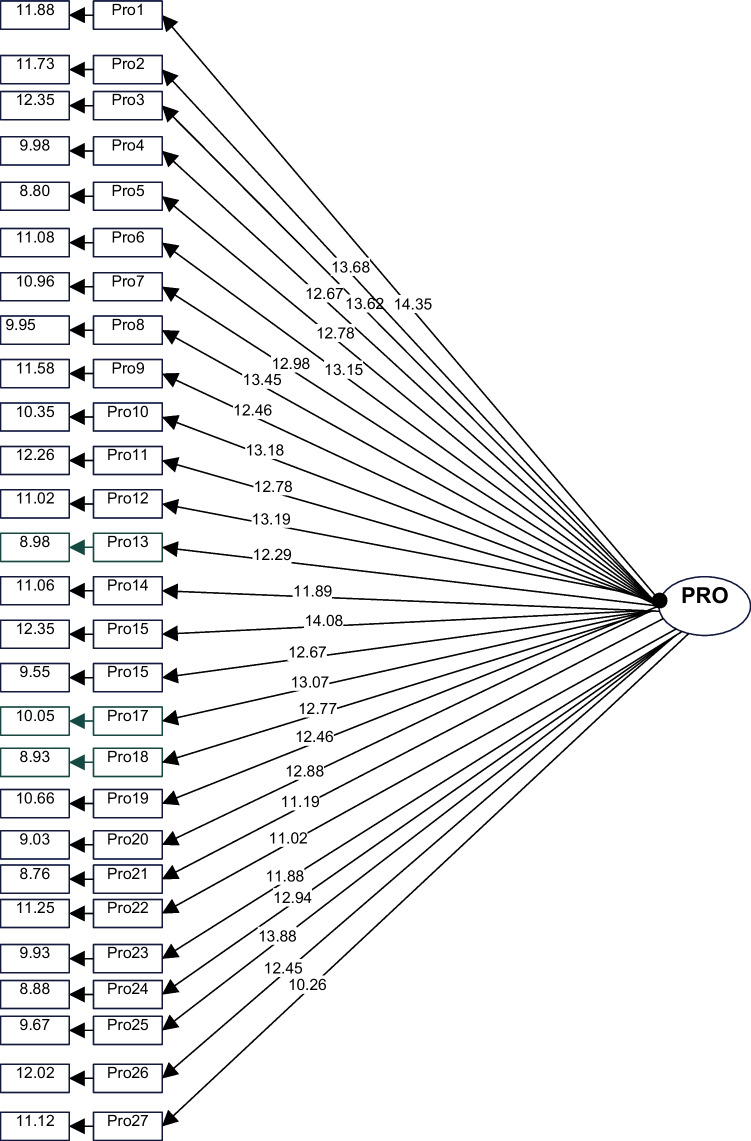
CFA for academic procrastination (*t*-values). Chi-square = 660.96, *df* = 324, *p*-value < 0.0001, RMSEA = 0.053

**Fig. 6:**
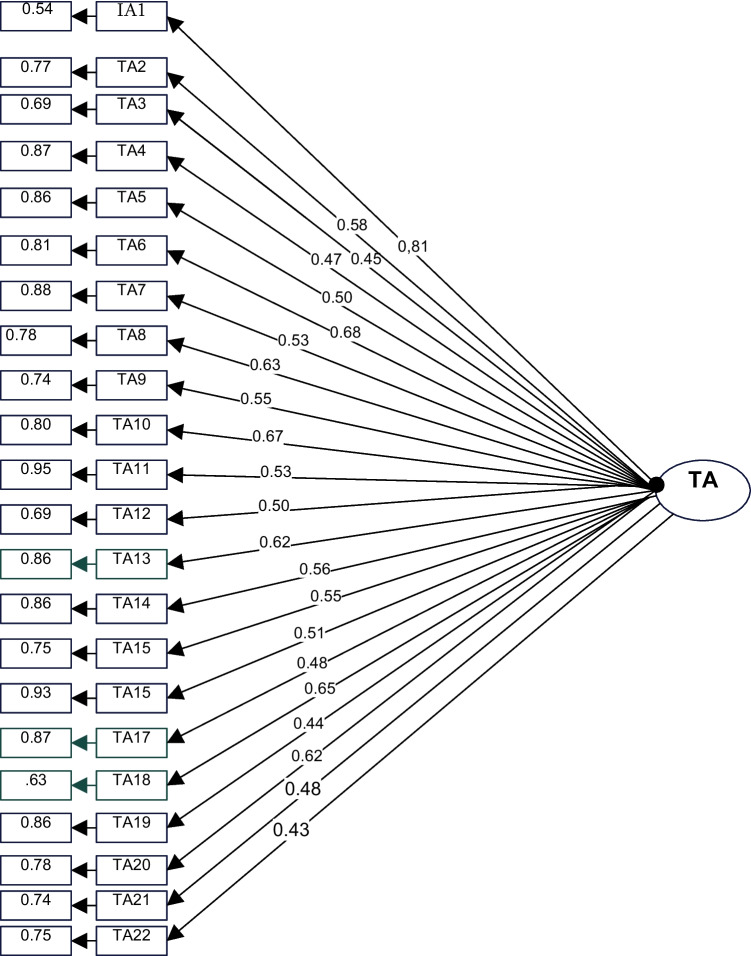
CFA for tolerance for ambiguity (standard solution). Chi-square = 267.52, *df* = 209, *p*-value < 0.0001, RMSEA = 0.041

**Fig. 7:**
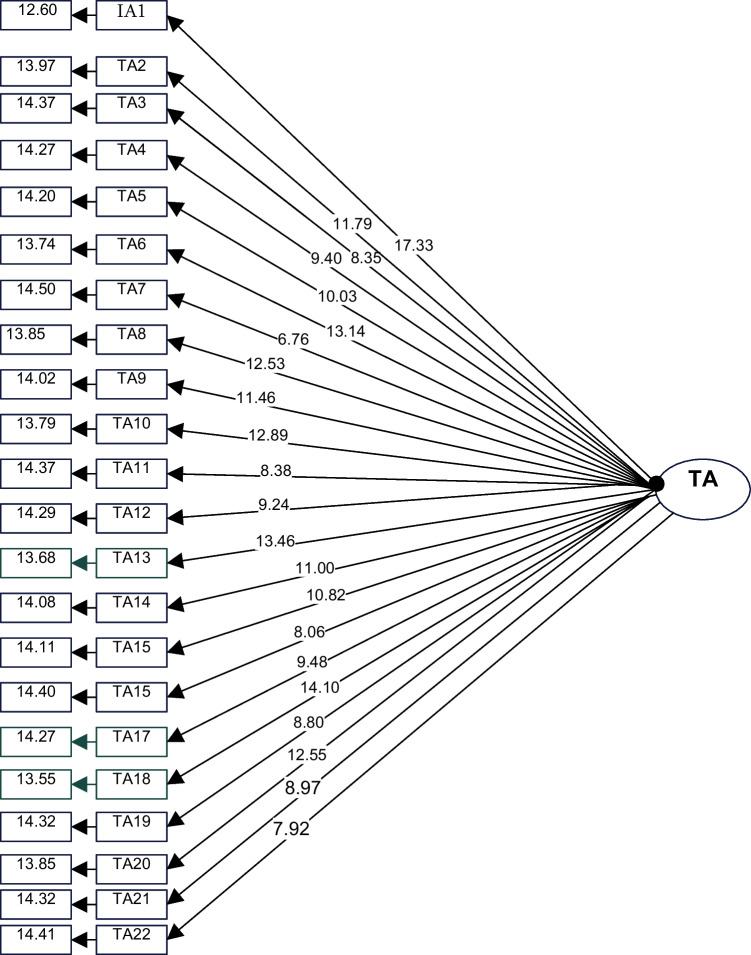
CFA for tolerance for ambiguity (*t*-values). Chi-square = 267.52, *df* = 209, *p*-value < 0.0001, RMSEA = 0.041

**Fig. 8:**
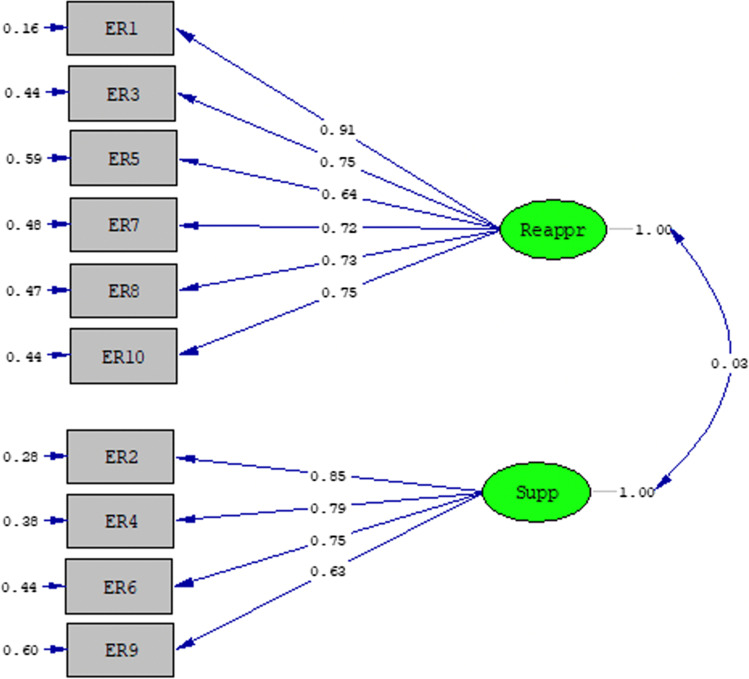
CFA for emotion regulation (standard solution). Chi-square = 50.66, *df* = 34, *p*-value-0.00028, RMSEA = 0.042

**Fig. 9:**
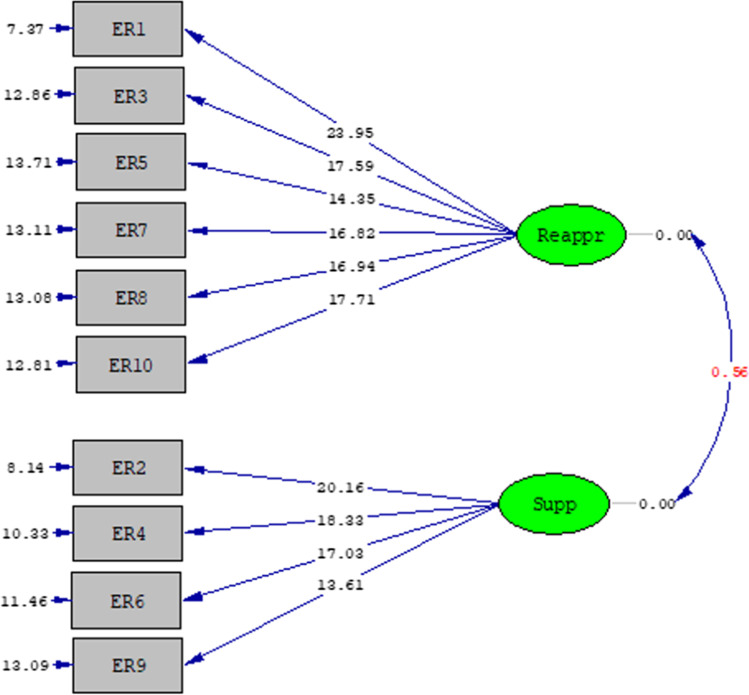
CFA for emotion regulation (*t*-values). Chi-square = 50.66, *df* = 34, *p*-value-0.00028, RMSEA = 0.042
